# The Dual Role of A2aR in Neuroinflammation: Modulating Microglial Polarization in White Matter Lesions

**DOI:** 10.1523/ENEURO.0579-24.2025

**Published:** 2025-03-04

**Authors:** Chang Cheng, Wenchao Cheng, Yuhan Wang, Xiuying Chen, Lan Zhang, Yi Li, Fa Shen, Dezhi Yuan, Pian Hong, Wen Huang

**Affiliations:** ^1^Department of Neurology, Xinqiao Hospital, Army Medical University (Third Military Medical University), Chongqing CN 400037, China; ^2^Department of Neurology, Chongqing Emergency Medical Center, Chongqing University Central Hospital, Chongqing CN 400014, China; ^3^Department of Neurology, The First Affiliated Hospital of Zhengzhou University, Zhengzhou, Henan CN 450052, China; ^4^Department of Neurology, Nanchong Central Hospital, Nanchong, Sichuan CN 634700, China; ^5^Department of Endocrinology, Xinqiao Hospital, Army Medical University (Third Military Medical University), Chongqing CN 400037, China

**Keywords:** A2aR, A2aR–mGluR5 heteromer, chronic cerebral hypoperfusion, microglia, neuroinflammation

## Abstract

Neuroinflammation has been widely recognized as the primary pathophysiological mechanism underlying ischemic white matter lesions (IWML) in chronic cerebral hypoperfusion (CCH). Adenosine A2A receptor (A2aR), an important adenosine receptor, exhibits a dual role in neuroinflammation by modulating both proinflammatory and anti-inflammatory responses. This study aimed to investigate the specific functions and mechanisms of A2aR in neuroinflammation. The findings revealed that A2aR initially exerted a proinflammatory role in the CCH model, transitioning to an anti-inflammatory role in later stages by regulating the phenotypic transformation of microglia. Further analyses using coimmunoprecipitation couple with mass spectrometry, in situ proximity ligation assay, AlphaFold protein structure prediction, [^35^S]GTPγS binding assay, and NanoBiT technology demonstrated that A2aR formed heteromers with mGluR5 during the early stage of CCH under high glutamate conditions, promoting the polarization of microglia toward a proinflammatory phenotype. In contrast, during later stages characterized by low glutamate levels, A2aR predominantly existed as a monomer, facilitating microglial polarization toward an anti-inflammatory phenotype. Our findings indicate that elevated glutamate levels drive the formation of A2aR–mGluR5 heteromers, contributing to neuroinflammation by promoting proinflammatory microglial polarization in CCH white matter. Conversely, under low glutamate conditions, A2aR primarily functions in its monomeric form, favoring an anti-inflammatory microglial phenotype and exerting a protective effect. This study elucidates the mechanism by which A2aR mediates microglial phenotypic transformation and participates in neuroinflammation under CCH. It also identifies A2aR as a potential therapeutic target for the treatment of IWML.

## Significance Statement

Neuroinflammation of white matter is widely recognized as the primary pathophysiological mechanism associated with vascular dementia. Adenosine A2A receptor (A2aR), a crucial adenosine receptor, exhibits a dual role in neuroinflammation by modulating both proinflammatory and anti-inflammatory responses. The objective of this study is to investigate the specific functions and mechanisms of A2aR in neuroinflammation. The results indicate that elevated glutamate levels facilitate the formation of A2aR–mGluR5 heteromers, thereby promoting the polarization of microglia toward a proinflammatory phenotype, which contributes to neuroinflammation in chronic cerebral hypoperfusion white matter. Conversely, under conditions of low glutamate, A2aR predominantly exists in monomeric form, which favors the polarization of microglia toward an anti-inflammatory phenotype, thereby exerting a protective effect.

## Introduction

Extensive lesions in the small arteries of the brain parenchyma, or stenosis of large cervical arteries and their intracranial branches, reduce blood flow to the deep white matter of the cerebral hemispheres, resulting in persistent cerebral insufficiency known as chronic cerebral hypoperfusion (CCH; Hou et al., 2015; Masdeu 2020; Takeda et al., 2020). CCH induces leukoaraiosis, also referred to as ischemic white matter lesions (IWML), a primary pathological condition associated with vascular cognitive impairment and vascular dementia in middle-aged and elderly individuals (Takeda et al., 2020). Effective interventions for IWML remain limited due to an incomplete understanding of its pathogenesis (Qin et al., 2020).

The pathophysiological mechanism of chronic hypoperfusion-induced white matter lesions is highly complex, involving energy dysregulation, excitatory amino acid toxicity, oxidative stress-induced damage, inflammatory responses, and apoptosis. Previous studies have demonstrated glial cell activation and proliferation, infiltration of inflammatory cells, elevated inflammatory mediator levels, and exacerbation of neuroinflammation in the white matter region affected by chronic hypoperfusion. Increasing evidence supports neuroinflammation as a primary underlying mechanism (Rajeev et al., 2022; Shkirkova et al., 2024).

Microglia, the primary mediators of neuroinflammation, exhibit diverse and complex roles in inflammatory injury (Zhang et al., 2017; Yang et al., 2023). Studies have shown that microglia promote inflammatory responses and exacerbate injury in cerebral ischemia (Zhou et al., 2018). Conversely, they play a crucial role in promoting oligodendrocyte differentiation, maturation, and the regeneration of white matter axons (Hagemeyer et al., 2017). These dual roles are attributed to microglial heterogeneity. Microglia can be activated via two main pathways, classical activation (M1) and alternative activation (M2), which mediate distinct functions. Although recent advancements in single-cell sequencing and related technologies have raised concerns about the oversimplification of M1/M2 classification, these phenotypes remain useful for broadly distinguishing microglia as proinflammatory (M1) or anti-inflammatory (M2) based on their general functions. While microglial phenotypic transformation has been implicated in various disease models, its role and mechanism in chronic hypoperfusion-induced white matter injury remain unclear and require further investigation.

Adenosine, an intermediate product in ATP synthesis and metabolism, is maintained at low levels under physiological conditions through efficient inactivation mechanisms. However, during stress conditions such as inflammation, ischemia, and hypoxia, adenosine concentrations increase rapidly (Schadlich et al., 2023). This surge initiates adenosine signaling, regulating neuroinflammation and other pathophysiological processes (Wang and Venton, 2019). Adenosine receptors, a class of G-protein–coupled receptors (GPCRs), mediate these processes by binding to specific receptor subtypes and activating associated signaling pathways (Peleli et al., 2017). These receptors are key pharmacological modulators of immune cell activity (Peleli et al., 2017; Mei et al., 2024). Among them, the adenosine A2A receptor (A2aR) has been implicated in central neuroinflammation due to its high affinity for adenosine and substantial brain expression (Fernandez et al., 2023). However, A2aR activation is generally associated with anti-inflammatory effects (Ahmad et al., 2017; Ganesana and Venton, 2018). In our investigation, we employed a microglia/oligodendrocyte coculture model under conditions of low glucose and hypoxia and observed that an A2aR agonist mitigated the inflammatory damage to oligodendrocytes induced by microglial activation (Huang et al., 2018). Nevertheless, numerous studies have reported that A2aR activation exacerbates neuroinflammatory injury (Chen et al., 2018; Colella et al., 2018; Cheng et al., 2022).

Interestingly, research in brain trauma models has shown that under varying glutamate concentrations, A2aR activation can engage either protein kinase A (PKA) or protein kinase C (PKC) pathways, resulting in anti-inflammatory or proinflammatory effects, respectively (Dai et al., 2010; Fu et al., 2019). These findings suggest a potential “bidirectional effect” of A2aR in neuroinflammation.

The configuration of A2aR may determine its function, with the bidirectional effect potentially linked to configuration changes under different injury conditions. Previous studies suggest that A2aR exerts synergistic or antagonistic effects primarily by forming heteromers with other GPCRs (Gaitonde and Gonzalez-Maeso 2017). The formation of these heteromers could influence the role of A2aR in the inflammatory response (Borroto-Escuela et al., 2018).

However, it remains unclear whether the “bidirectional effect” of A2aR in CCH-induced neuroinflammation is related to the formation of heteromers. This study aims to elucidate the specific role and mechanism of A2aR in neuroinflammation under CCH.

## Materials and Methods

### Animals

Sprague Dawley (SD) rats were obtained from the Animal Experiment Center of the Third Military Medical University. All rats were maintained in pathogen-free conditions with a 12 h light/dark cycle, an ambient temperature of 22 ± 2°C, and *ad libitum* access to food and water. Ethical approval for all animal experiments was granted by the Laboratory Animal Welfare and Ethics Committee of the Third Military Medical University (Ethical Approval Number: AMUWEC20210276), and the experiments complied with relevant regulations and guidelines. All methods adhered to the Animal Research: Reporting of In Vivo Experiments (ARRIVE) guidelines.

This study comprised three parts and involved 146 12-week-old male SD rats. In the first part, five rats underwent bilateral common carotid artery occlusion (BCCAO) surgery to establish CCH model. Doppler flowmeter was used to measure cerebral blood flow (CBF) in rats to verify the effect and stability of the model.

In the second part, 72 rats were randomly divided into three groups, each further subdivided into four subgroups (1, 2, 3, and 4 W). The three groups were the SH group, the CCH group, and the CGS21680 group. The CGS21680 group received CGS21680 (0.1 mg/kg, i.p.; Borroto-Escuela et al., 2019) on Days 1–7, 8–14, 15–21, and 22–28 postoperation for the 1, 2, 3, and 4 W subgroups, respectively. The other groups received equal volumes of dimethyl sulfoxide (DMSO; Fig. 2*A*).The rats in each subgroup were killed under excessive anesthesia after the last drug (or DMSO) treatment. Three rats in each subgroup were randomly selected to prepare paraffin sections for immunofluorescent staining and LBF staining. Fresh brain tissues were obtained from the other three rats for cytokine and protein expression measurement.

In the third part, nine rats were divided into the SH group, CCH group, and riluzole group to measure glutamate concentrations in cerebral microdialysates at different time points.

In the last part, 60 rats were randomly divided into five groups, each further subdivided into two subgroups (1 week and full-dose subgroups): A, SH group; B, CCH group; C, CCH + A2aR agonist group; D, CCH + riluzole group; and E, CCH + A2aR agonist + riluzole group. Groups C and E received daily CGS21680 (0.1 mg/kg, i.p.) postoperation. Groups D and E received daily riluzole (5 mg/kg, i.p.; Citraro et al., 2023), while other groups received equal volumes of DMSO (Fig. 8*A*). Tissues were sampled in the same manner and at the same time points as in the second part.

### CCH model

BCCAO was performed to induce long-term reductions in CBF and white matter damage. Rats were anesthetized with 1% pentobarbital sodium (50 mg/kg, i.p.), and the left common carotid artery was carefully exposed and ligated with a surgical silk suture. After 30 min, the right common carotid artery was similarly ligated. Sham-operated rats underwent identical procedures without arterial ligation. A heating pad was used to maintain body temperature during the surgery.

### CBF measurement

The CBF of rats was measured by PeriFlux System 5000 laser Doppler flowmeter (Perimed, RRID: SCR_015962) with a small straight probe 407-1, as described previously (Wang et al., 2024). After being anesthetized, the rats were secured to the stereotaxic apparatus. The skull was exposed, and then the examined region was thinly polished. The tip of the probe was positioned vertically over the skull using a probe holder (1 mm posterior and 5 mm lateral from the bregma). The CBF was continuously monitored and recorded using laser Doppler flowmeter.

### Luxol fast blue (LFB) staining

After deep anesthesia, rats were perfused transcardially with phosphate-buffered saline (PBS) followed by 4% paraformaldehyde. Brains were removed and fixed in 4% paraformaldehyde for 24 h, dehydrated, and embedded in paraffin for sectioning into 10 μm slices. Sections were stained using the LFB staining kit (G1030, ServiceBio) following the manufacturer's instructions and observed under a microscope.

LFB staining scores were used to assess myelinated nerve fiber damage. Three nonoverlapping fields in the corpus callosum were selected as regions of interest, and lesion severity was graded on a five-point scale: Grade 0 (normal), Grade 1 (disarranged nerve fibers), Grade 2 (cleft formation in myelinated fibers), Grade 3 (marked vacuoles), and Grade 4 (loss of myelinated fibers). Independent investigators blinded to the groupings conducted the assessments, and average values were calculated to quantify demyelination.

### Immunofuorescence

The brain tissue was collected and processed as described for LFB staining. After fixation in 4% paraformaldehyde, 10 μm coronal sections were prepared. Antigen retrieval was performed based on the antibody requirements and tissue characteristics, followed by blocking with 3% bovine serum albumin (BSA) for 30 min. Sections were incubated overnight at 4°C with primary antibodies, followed by secondary antibodies conjugated with fluorescent tags for 50 min at room temperature in a dark environment. 4′,6-diamidino-2-phenylindole (DAPI) was applied for 5 min before observation under a fluorescent microscope.

Multiple immunofluorescent staining was conducted using tyramide signal amplification. After antigen retrieval and blocking with 3% hydrogen peroxide (H_2_O_2_) and 3% BSA, sections were incubated overnight at 4°C with the primary antibody, followed by incubation with horseradish peroxidase (HRP)-labeled secondary antibodies for 50 min in a dark environment. Tyramide labeled with fluorescent dye was then applied for 10 min. This process was repeated for the second and third sets of primary and secondary antibodies. After DAPI staining, sections were observed under a fluorescent microscope.

Primary antibodies: mouse anti-iba-1 (1:3,000, GB12105, ServiceBio), rabbit anti-Arg-1 [1:100, 93668S, Cell Signaling Technology (CST)], rabbit anti-iNOS (1:100, ab3523, Abcam), rabbit anti-myelin basic protein (MBP; 1:100, AF4085, Affinity), mouse anti-mGluR5 (1:1,000, MA5-2769, Invitrogen), rabbit anti-A2aR (1:100, PA1-042, Invitrogen).

Secondary antibodies: Alexa Fluor 568-conjugated goat anti-mouse IgG H&L (1:500, ab175473, Abcam), Alexa Fluor 647-conjugated goat anti-rabbit IgG H&L (1:500, ab150079, Abcam), Alexa Fluor 647-conjugated goat anti-mouse IgG H&L (1:500, ab150115, Abcam), Alexa Fluor 488-conjugated goat anti-rabbit IgG H&L (1:500, ab150077, Abcam), and DAPI (G1012, ServiceBio).

### Primary microglia

Brains were harvested from 10 neonatal SD rats (Postnatal Days 1–3) after careful removal of the meninges. The brain tissue was dissociated through 0.25% trypsin digestion at 37°C for 30 min, followed by mechanical grinding and filtration through a 40 μm cell strainer. The cells were centrifuged at 2,000 × *g* for 10 min, resuspended, and inoculated into 75 cm^2^ polylysine-coated culture flasks containing 10% heat-inactivated fetal bovine serum (FBS; Hyclone) and 100 U penicillin/100 μg streptomycin (Invitrogen). The cells were incubated at 37°C in 95% air/5% CO_2_ for 12–14 d until the cultures reached confluence. Microglia were separated from glial cell cultures by shaking the flasks at 200 rpm at 37°C for 2 h. Suspended microglia were collected, inoculated into a six-well plate, adhered for 20 min, and incubated overnight for subsequent experiments.

### Chronic hypoxia cell model

As described previously (Zhang et al., 2020), primary microglia were seeded on six-well cell culture plates and cultured in MEM medium with 10% FBS and 1% penicillin/streptomycin. Cells were transferred into a three-gas cell incubator set at 37°C with 3% O_2_, 5% CO_2_, and 92% N_2_ and cultured for 24 h to establish a chronic hypoxia cell model, simulating microglial survival under CCH conditions. In the first part, cells were divided into six groups:

1. A: Blank control group
2. B: Chronic hypoxia group
3. C: Glutamate 20 μM group
4. D: Glutamate 40 μM group
5. E: Chronic hypoxia + glutamate 20 μM group
6. F: Chronic hypoxia + glutamate 40 μM group

Groups B–F were exposed to chronic hypoxia. Groups C and E were incubated with glutamate (20 μM), Groups D and F were incubated with glutamate (40 μM), while Groups E and F were treated with CGS21680 (100 nM; Fu et al., 2019).After the cells in each group were treated for 24 h according to the above conditions, the cell supernatant and cells were collected respectively. The cytokine levels of the cell supernatant were determined by enzyme-linked immunosorbent assay (ELISA). The cells were lysed on ice after washing with PBS for subsequent PKA/PKC activity measurement and Western blot (WB).

In the second part, cells were divided into six groups:

1. **A**: Blank control group
2. **B**: Chronic hypoxia group
3. **C**: Chronic hypoxia + A2aR agonist group
4. **D**: Chronic hypoxia + A2aR agonist + PKA inhibitor group
5. **E**: Chronic hypoxia + glutamate + A2aR agonist group
6. **F**: Chronic hypoxia + glutamate + A2aR agonist + PKC inhibitor group

Groups B–F were exposed to chronic hypoxia. Groups C–F were incubated with CGS21680 (100 nM; Fu et al., 2019), while Group D included H89 (10 μM; Deng et al., 2024), Groups E–F were treated with glutamate (40 μM), and Group F included Go 6983 (10 μM; Shen et al., 2019). Subsequent processing was the same as for the first part.

### ELISA

TNF-α, IL-1β, and IL-10 concentrations in white matter and primary microglia supernatant were measured using Rat TNF-α ELISA Kit (EK382, MultiSciences Biotech), Rat IL-1β ELISA Kit (EK301B, MultiSciences Biotech), and Rat IL-10 ELISA Kit (EK310, MultiSciences Biotech) according to the manufacturer's instructions.

### PKA and PKC activity assay

PKA and PKC activities in primary microglia were assessed using the PKA Kinase Activity Assay Kit (ab139435, Abcam) and PKC Kinase Activity Assay Kit (ab139437, Abcam). Briefly, cells were washed with PBS and lysed with buffer containing 50 mM KH_2_PO_4_, 1.5 mM MgCl_2_, 10 mM NaCl, and 1 mM EDTA, pH 7.4, on ice. Lysates were collected by scraping, centrifuged at 18,000 × *g* for 15 min at 4°C, and diluted 1:20 in the assay kit's dilution buffer. Diluted samples were added to a 96-well plate provided with the kit, and subsequent steps followed the manufacturer's protocol. Protein concentrations were measured using the BCA assay, and PKA/PKC activities were normalized to protein levels.

### Cerebral microdialysis

Rats were anesthetized with 10% chloral hydrate (0.34 ml/100 g, i.p.), and 1 ml lidocaine was injected into the scalp for infiltration anesthesia. After the parietal bone was exposed, a 22 gauge stainless steel cannula (external diameter, 0.7 mm) was implanted into the right ventricle (posterior fontanel 0.5 mm, anterior fontanel midline right 1 mm, perpendicular depth 2.5 mm). The cannula was secured with dental cement, and the skull was protected with the surrounding tissue. After 12 h of recovery, a microdialysis probe was placed in the cannula. Samples were collected every 30 min at a flow rate of 1 μl/min using an injection pump and stored at −80°C for further analysis.

### HPLC-MS

Glutamate concentrations in cerebral microdialysates were measured using high-performance liquid chromatography coupled with mass spectrometry (HPLC-MS). Chromatographic separation was performed on a Waters XBridge C18 column (100 × 3 mm, 3.5 μm). The mobile phase consisted of the following:

1. **A**: 0.05 mol/L ammonium acetate in water
2. **B**: Methanol:acetonitrile:water (20:60:20, *v*/*v*/*v*)

Gradient elution was conducted at 0.3 ml/min with the following program:

1. 0–10 min: Linear increase from 10% B to 37% B
2. 10–12 min: Increase from 37% B to 90% B
3. 12–15 min: Stabilization at 20% B

Mass spectrometry parameters were as follows:

- **ESI source settings**: Spray voltage 4,000 kV, sheath gas 50 Arb, auxiliary gas 15 Arb
- **Mass spectrometry settings**: SIM scanning mode, S-lens 60%, quadrupole isolation window 2 Da, Orbitrap resolution 60,000, scan range 200–1,000

Adenosine concentrations in cerebral microdialysates were also measured using HPLC-MS. Chromatographic separation was performed on a Zorbax SB-Aq C18 column (250 × 4.6 mm, 5 μm). The mobile phase consisted of the following:

1. **A**: Formic acid aqueous solution (containing 0.01% formic acid)
2. **B**: Methanol

Gradient elution was conducted at 0.6 ml/min with the following program:

1. 0–1 min: 2% B
2. 1–3.5 min: 2% to 95% B
3. 3.5–4.5 min: 95%
4. 4.5–4.6min: 95% to 2% B
5. 4.6–∼5.5 min: 2% B。

Mass spectrometry parameters were as follows:

- **ESI source settings**: Negative ion mode, quantitative analysis was performed using multiple reactive ion monitoring mode (MRM). Spray voltage 5,500 kV, sheath gas 60 Arb, auxiliary gas 40 Arb, decoupling voltage −30 V.
- **Mass spectrometry settings**: quantitative analysis was performed using MRM. Adenosine was measured at m/z 268.2→137.2.

### NanoBiT

Recombinant fusion protein vectors, incorporating NanoLuc luciferase fragments LgBit and SmBit fused to A2aR, mGluR5, and PNCA (proliferating cell nuclear antigen), were constructed using standard molecular biology techniques. These recombinant vectors were designed for expression in a eukaryotic system. The construction and sequencing of the vectors were performed by Chongqing Jinmai Biotechnology. Receptive cells were prepared from blank *Escherichia coli* strains, and the recombinant plasmids were introduced into the receptive cells via heat shock. Plasmids were purified using the Plasmid Maxprep Kit (N001, Vigorous Biotechnology).

HEK293ET cells were seeded into 96-well plates at a density of 1.8 × 10^4^ cells per well and cultured in MEM medium with 10% FBS and 1% penicillin/streptomycin for 24 h. Recombinant plasmids were transfected into HEK293ET cells using the Lipofectamine 3000 Transfection Reagent Kit (L3000150, Thermo Fisher Scientific). After 48 h, the cells were washed, and the Nano-Glo Live Cell Reagent (Promega) was added following the Nano-Glo Live Cell Assay System protocol. Luminescence signals were measured using an Enspire multifunctional enzyme marker.

### AlphaFold 3 (simulation of protein interaction)

AlphaFold3 was installed via pip and run on a server following Abramson J's protocol (Abramson et al., 2024). The amino acid sequences of the target proteins were input, and default parameters were applied. Interactions between (1) A2aR and mGluR5, (2) A2aR and Gs, (3) A2aR and Gq, (4) A2aR–mGluR5 heteromer and Gs, ND (5) A2aR–mGluR5 heteromer and Gq were simulated, yielding protein complex crystals. Using the Protein Preparation Wizard module in the Schrödinger software, the crystal structures were preprocessed, native ligand states were regenerated, H-bond assignments were optimized, protein energy was minimized, and water molecules were removed. Different chains in the protein–protein interaction complex were labeled with unique colors, and the surface of the proteins was rendered to create a 3D stereoscopic view. The Protein Interaction Analysis module identified specific binding regions.

### Cell membrane preparation

Membrane preparations were done as previously described (Gerbier et al., 2021). Briefly, primary microglia were seeded on BeyoGold six-well cell culture plates at a density of 1.8 × 10^4^ cells per well. After treatment according to the experimental conditions (see above, Chronic hypoxia cell model, for details), cells were mechanically detached in 10 ml PBS with a scraper and harvested in precooled 50 ml Falcon tubes. Then, cells were resuspended in 10 ml/10^6^ cells of ice-cold buffer containing 5 mM Tris, 2 mM EDTA, pH 7.4, 5 mg/l soybean trypsin inhibitor, 5 mg/l leupeptin, and 10 mg/l benzamidine. Cell suspensions were homogenized with a Polytron Homogenizer (Janke & Kunkel Ultra-Turrax T25) three times for 5 s each time at maximal setting. Lysates were centrifuged at 43,000 × *g* for 20 min at 4°C, and the pellet was washed once in the same buffer. The final pellet was resuspended in 75 mM Tris, pH 7.4, 5 mM MgCl2, 2 mM EDTA, and protease inhibitors (as above) and immediately used in [35S]GTPγS binding assay experiments. Protein concentrations were determined using the BCA Protein Assay Kit (P0012, Beyotime).

### [^35^s]GTPγS binding assay

A modified version of the previously described protocol was used (Noseda et al., 2021). Briefly, proteins (50 μg per tube) were incubated with the binding buffer (20 mM HEPES, 100 mM NaCl, 3 mM MgCl2), pH 7.4, and supplemented with 0.5 μM GDP for Gq and Gs and with 1 nM [^35^S]GTPγS and the indicated concentration of ligands (final volume of 200 μl). Nonspecific binding was determined in the presence of 10 μM GTPγS. The mix was incubated at 30°C for 60 min, and reactions were stopped by the addition of ice-cold binding buffer. The samples were centrifuged at 15,000 × *g* for 15 min at 4°C, and the resulting pellets were carefully resuspended in detergent buffer (100 mM Tris–HCl, 200 mM NaCl, 1 mM EDTA, 1,5% Igepal CA-630), and pH 7.4, supplemented with 0.2% SDS, then rotated at 4°C for 120 min, and precleared with Pansorbin (Calbiochem). After centrifugation at 15,000 × *g*, the supernatant was incubated with protein A/G beads (Santa Cruz Biotechnology) and antibody against Gq or Gs (Santa Cruz Biotechnology, sc-136181 and sc-135914, respectively), overnight at 4°C. The immunocomplexes were washed twice with detergent buffer, and bound [^35^S]GTPγS was measured by liquidscintillation spectrometry.

### In situ proximity ligation assay (in situ PLA)

Cells were seeded on 1 cm glass slides, precooled with PBS, and fixed with 4% formaldehyde at 4°C for 10 min. Duolink blocking solution (DUO82040, Sigma-Aldrich) was added to each sample. Primary antibodies, diluted with Duolink antibody thinner, were incubated overnight with the samples. Subsequently, PLA probes were added per the Duolink PLA Probe PLUS (DUO92004, Sigma-Aldrich) and MINUS (DUO92002, Sigma-Aldrich) protocols. Finally, Duolink in situ sealant containing DAPI (DUO82040, Sigma-Aldrich) was applied to the slides, which were observed under a confocal microscope.

### Co-IP–MS

Coimmunoprecipitation (Co-IP) was conducted using the Beaver Beads Protein A/G Immunoprecipitation Kit (Beaver Biomedical Engineering). Proteins from primary rat microglia were collected using a binding buffer supplemented with a 1× protease inhibitor cocktail. Pretreated magnetic beads (Protein A/G) were incubated with mGluR5/A2aR antibody or IgG antibodies at room temperature for 30 min. The bead–antibody complexes were then mixed with cell lysates and rotated overnight at 4°C. Supernatants were analyzed via SDS–PAGE and identified through MS by Wuhan GeneCreate Biological Engineering.

### WB

Total proteins were extracted from white matter and primary microglia using RIPA lysis buffer (P0038, Beyotime) and a protease/phosphatase inhibitor cocktail (P1046, Beyotime). Protein concentrations were determined using the BCA Protein Assay Kit (P0012, Beyotime). Proteins were separated via SDS–PAGE, transferred onto PVDF membranes (Merck Millipore), and blocked. Membranes were incubated with primary antibodies overnight at 4°C and subsequently with HRP-conjugated secondary antibodies. Bands were visualized using chemiluminescence.

Primary antibodies included the following:

1. Mouse anti-mGLUR5 (1:1,000, MA5–2769, Invitrogen)
2. Rabbit anti-A2aR (1:1,000, PA1-042, Invitrogen)
3. Rabbit anti-Arg-1 (1:1,000, 93668S, CST)
4. Rabbit anti-iNOS (1:1,000, ab3523, Abcam)
5. Phospho-cAMP response element-binding protein (CREB) rabbit mAb (1:1,000, #9198, CST)
6. NF-κB p65 rabbit mAb (1:1,000, #4764, CST)

Secondary antibodies included the following:

1. HRP-conjugated goat anti-mouse IgG (1:5,000, GAM007, LiankeBio)
2. HRP-conjugated goat anti-rabbit IgG (1:5,000, GAR007, LiankeBio)

### Statistical analysis

Data were expressed as mean ± SD or percentages. Shapiro–Wilk normality test assessed the normality of the all continuous variables. For normally distributed data, unpaired *t* tests compared differences between two groups, while one-way ANOVA with Tukey's post hoc test was used for multiple group comparisons. For data that did not follow a normal distribution, the Mann–Whitney *U* test compared differences between two groups, while the Kruskal–Wallis test was used for multiple group comparisons. The Kruskal–Wallis *H* test was used for ordinal data. Statistical significance was set at *p* < 0.05. Analyses were performed using GraphPad Prism 9.3 (GraphPad Software).

## Results

### Microglia-dominated neuroinflammation is an important pathological mechanism for the occurrence and development of CCH white matter lesion

**Figure 1.**
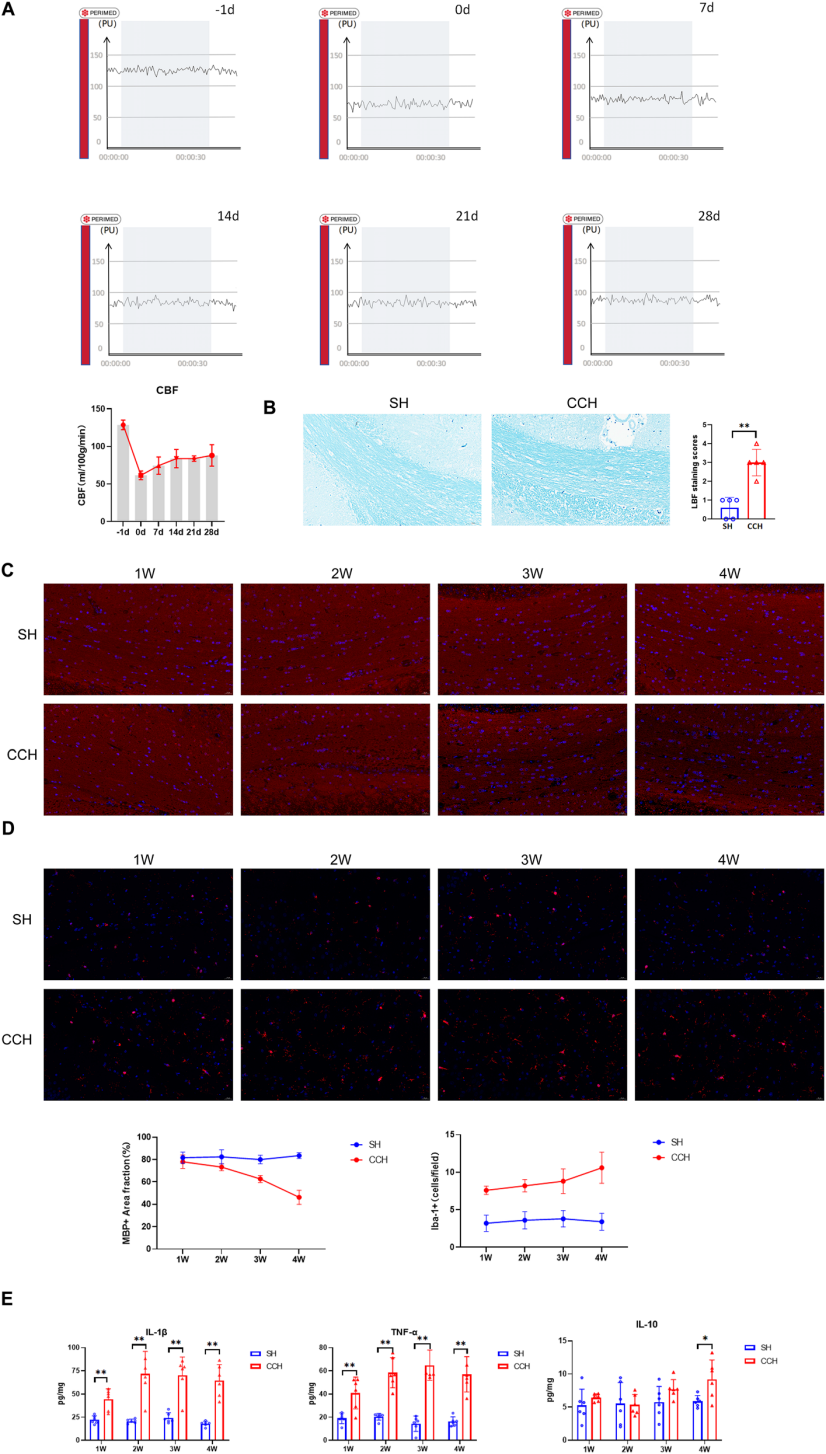
White matter neuroinflammation in a CCH rat model.

To investigate the impact of BCCAO on inducing WMLs in CCH group rats, we conducted laser Doppler CBF measurement to observe changes in CBF after BCCAO and performed LFB staining and MBP immunofluorescent staining to assess myelinated nerve fiber damage in the cerebral white matter area. The results of CBF measurement indicated that, in comparison with the −1 d (baseline level), the CBF values exhibited a significant decrease in the 7, 14, 21, and 28 d after surgery. From 7 to 28 d after surgery, the CBF values gradually recovered but remained significantly lower than those of the −1 d (baseline level; Fig. 1*A*). LBF staining and MBP immunofluorescent staining indicated that, in the CCH animal model, significant myelin and axon damages were observed in the white matter lesion area (corpus callosum; Fig. 1*B*,*C*). Proliferation and activation of microglia were evident in these regions (Fig. 1*D*).

These findings suggest that BCCAO is a reliable CCH model that can cause a steady decrease in CBF and white matter lesions. The results also showed that neuroinflammation is the primary pathological mechanism underlying CCH white matter lesions, with proinflammatory microglia serving as the main effector cells.

### A2ar plays a bidirectional role in CCH neuroinflammation

**Figure 2.**
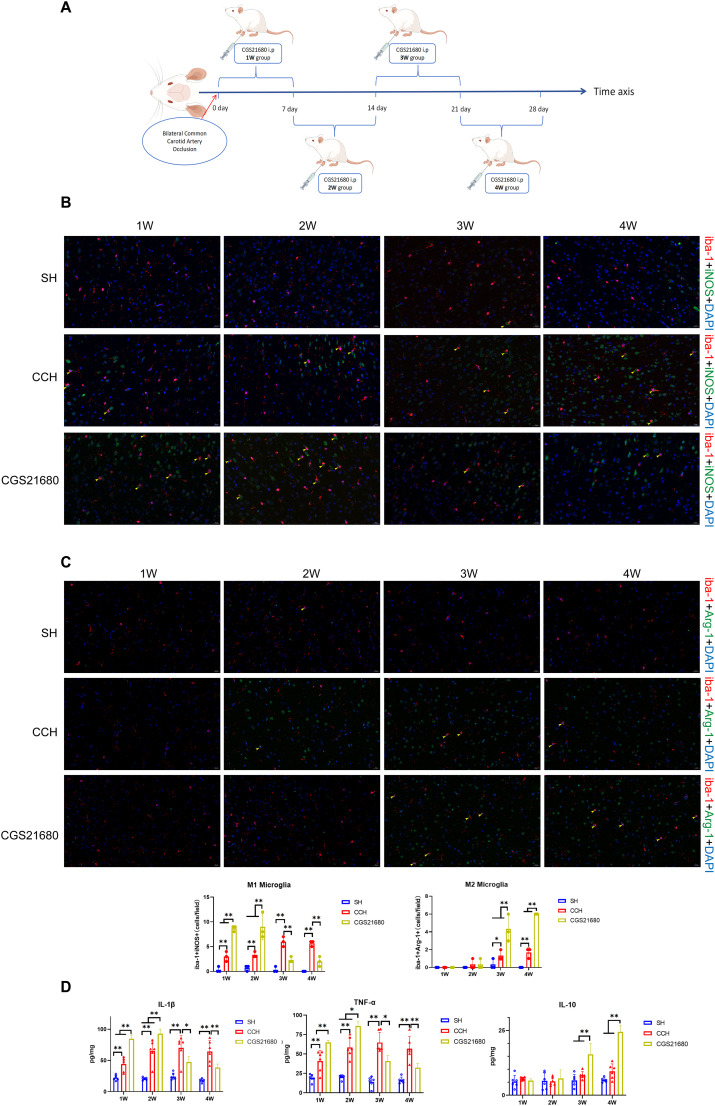
A2aR plays a bidirectional role in CCH neuroinflammation.

To explore the role of A2aR in the progression of CCH-induced neuroinflammation, we administered the A2aR agonist CGS21680 to CCH model rats during four distinct time periods: Days 1–7 (first week), Days 8–14 (second week), Days 15–21 (third week), and Days 22–28 (fourth week) postoperation (Fig. 2*A*).

ELISA results revealed that, compared with the SH group, the inflammatory cytokines IL-1β and TNF-α were significantly elevated at all time points, with no significant changes in the anti-inflammatory cytokine IL-10 (Fig. 2*D*). Compared with the CCH group, the inflammatory cytokines IL-1β and TNF-α were significantly elevated in the 1 and 2 W CGS21680 treatment subgroups, with no significant changes in the anti-inflammatory cytokine IL-10 (Fig. 2*D*). In contrast, in the 3 and 4 W treatment subgroups, IL-1β and TNF-α levels were markedly reduced, while IL-10 levels were significantly increased (Fig. 2*D*). These findings suggest that activating A2aR at different time points exerts distinct, even opposing, effects.

Immunofluorescent staining further demonstrated that, compared with the SH group, the number of iNOS and iba-1 double–positive cells was significantly increased at all time points in the CCH group (Fig. 2*B*), with no significant change in iba-1 and Arg-1 double–positive cells in the 1 and 2 W subgroups and increased in the 3 and 4 W subgroups (Fig. 2*C*). Compared with the CCH group, the number of iNOS and iba-1 double–positive cells was significantly increased in the 1 and 2 W subgroups treated with CGS21680 (Fig. 2*B*), with no significant change in iba-1 and Arg-1 double–positive cells (Fig. 2*C*). Conversely, compared with the CCH group, iNOS and iba-1 double–positive cells were significantly decreased in the 3 and 4 W subgroups treated with CGS21680 (Fig. 2*B*), while iba-1 and Arg-1 double–positive cells were significantly increased (Fig. 2*C*). These results indicate that CGS21680 administration during Weeks 1 and 2 accelerated the polarization of microglia toward the proinflammatory M1 phenotype, while administration during Weeks 3 and 4 inhibited M1 polarization and promoted polarization to the anti-inflammatory M2 phenotype. Collectively, these findings highlight the opposing roles of A2aR in neuroinflammation under the CCH model during different time periods.

### A2ar and mGluR5 interact to form heteromer in microglia

**Figure 3.**
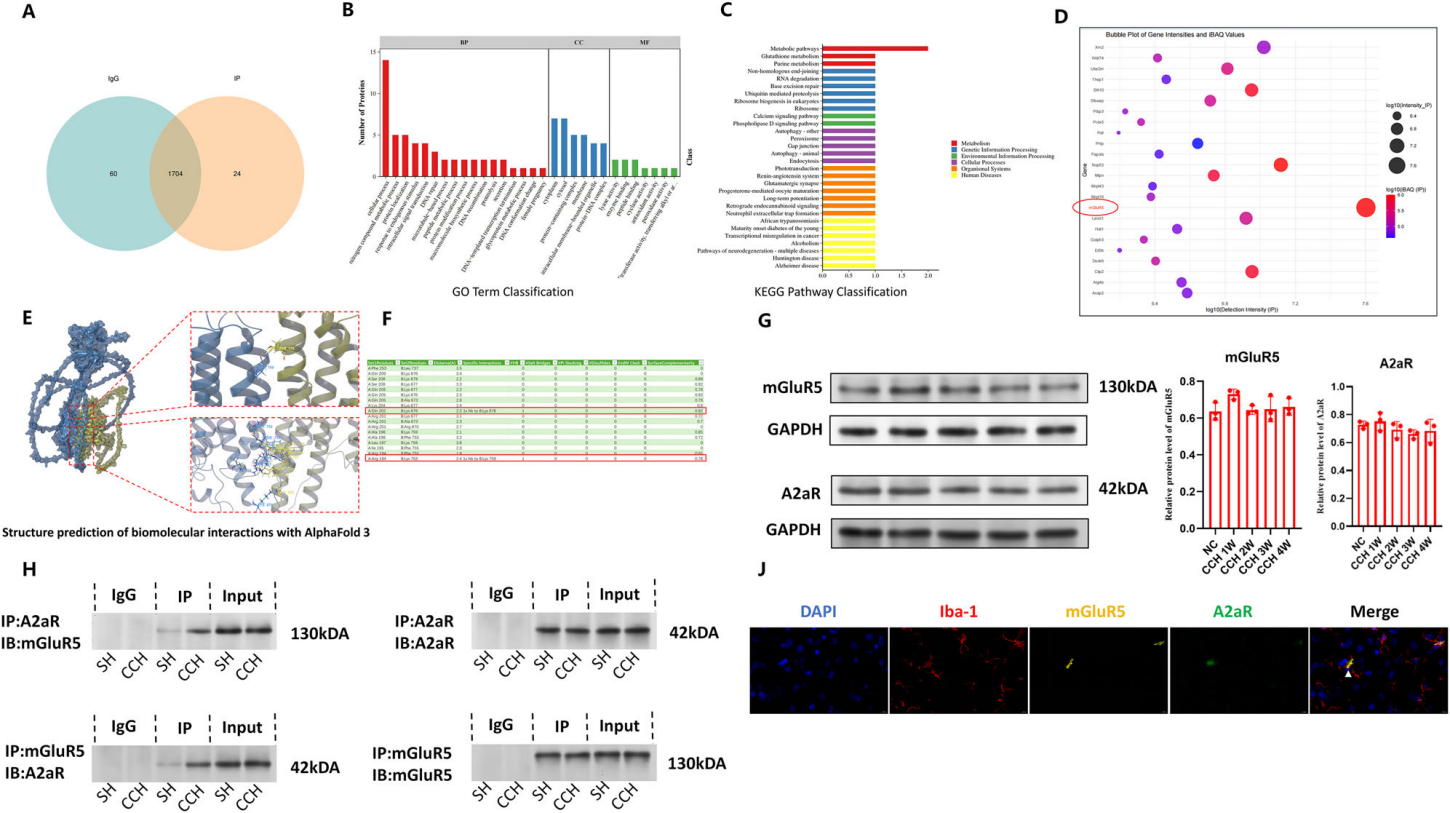
A2aR and mGluR5 interact to form heteromer in microglia.

Based on the principle that configuration determines function, we hypothesized that A2aR interacts with other molecules to alter its configuration, thereby influencing the phenotype of microglia and mediating its bidirectional effects in CCH. Using the Co-IP coupled with mass spectrometry (Co-IP–MS) method, we identified molecules interacting with A2aR through mass spectrometry and bioinformatics analyses. Immunoprecipitation of primary microglia treated with chronic hypoxia successfully isolated A2aR-binding proteins. Mass spectrometry identified 1,728 and 1,764 proteins in the IP and IgG groups, respectively, with 24 proteins unique to the IP group considered as potential A2aR-binding proteins (PBPs; Fig. 3*A*).

GO analysis revealed that PBPs were significantly enriched in the following categories:

1. **Biological Processes:** Cellular processes, nitrogen compound metabolic processes, and protein localization
2. **Cellular Component:** Cytoplasm, cytosol, membrane, and protein-containing complexes
3. **Molecular Function:** Lyase activity, enzyme binding, and peptide binding (Fig. 3*B*)

KEGG analysis indicated significant enrichment in metabolic pathways, glutathione metabolism, and glutamatergic synapses (Fig. 3*C*). Among the 24 PBPs, mGluR5 exhibited the highest signal strength and iBAQ value. As a GPCR and membrane protein, similar to A2aR, mGluR5 was identified as a likely interaction partner capable of altering A2aR configuration (Fig. 3*D*).

The protein expression of A2aR and mGluR5 were measured by WB, and the results showed that there was no significant difference in the expression of A2aR and mGluR5 at each time point from 1 to 4 W compared with the blank group (representing the baseline level; Fig. 3*G*). The results suggest that the functional changes of A2aR at different periods in the CCH model may not be induced by the protein expression changes but may involve changes in configuration or stability.

Using AlphaFold3, we predicted the protein structures of mGluR5 and A2aR. TYR175, GLN202, and ARG194 of A2aR were found to form hydrogen bonds with VAL788, LYS676, and LYS759 of mGluR5, respectively (Fig. 3*E*). These findings suggest a strong propensity for A2aR and mGluR5 to interact and form stable heteromers (Fig. 3*F*).

The presence of A2aR–mGluR5 heteromers was further validated through Co-IP and immunofluorescent analyses. Co-IP results demonstrated that, in primary microglia treated with chronic hypoxia, mGluR5 bands were coimmunoprecipitated by the anti-A2aR antibody, while A2aR bands were coimmunoprecipitated by the anti-mGluR5 antibody (Fig. 3*H*). Immunofluorescent staining confirmed the colocalization of iba-1, A2aR, and mGluR5 in the white matter of CCH rats (Fig. 3*J*). These findings conclusively demonstrate the formation of A2aR–mGluR5 heteromers in microglia.

### High glutamate concentration promotes the formation of A2aR–mGluR5 heteromer

**Figure 4.**
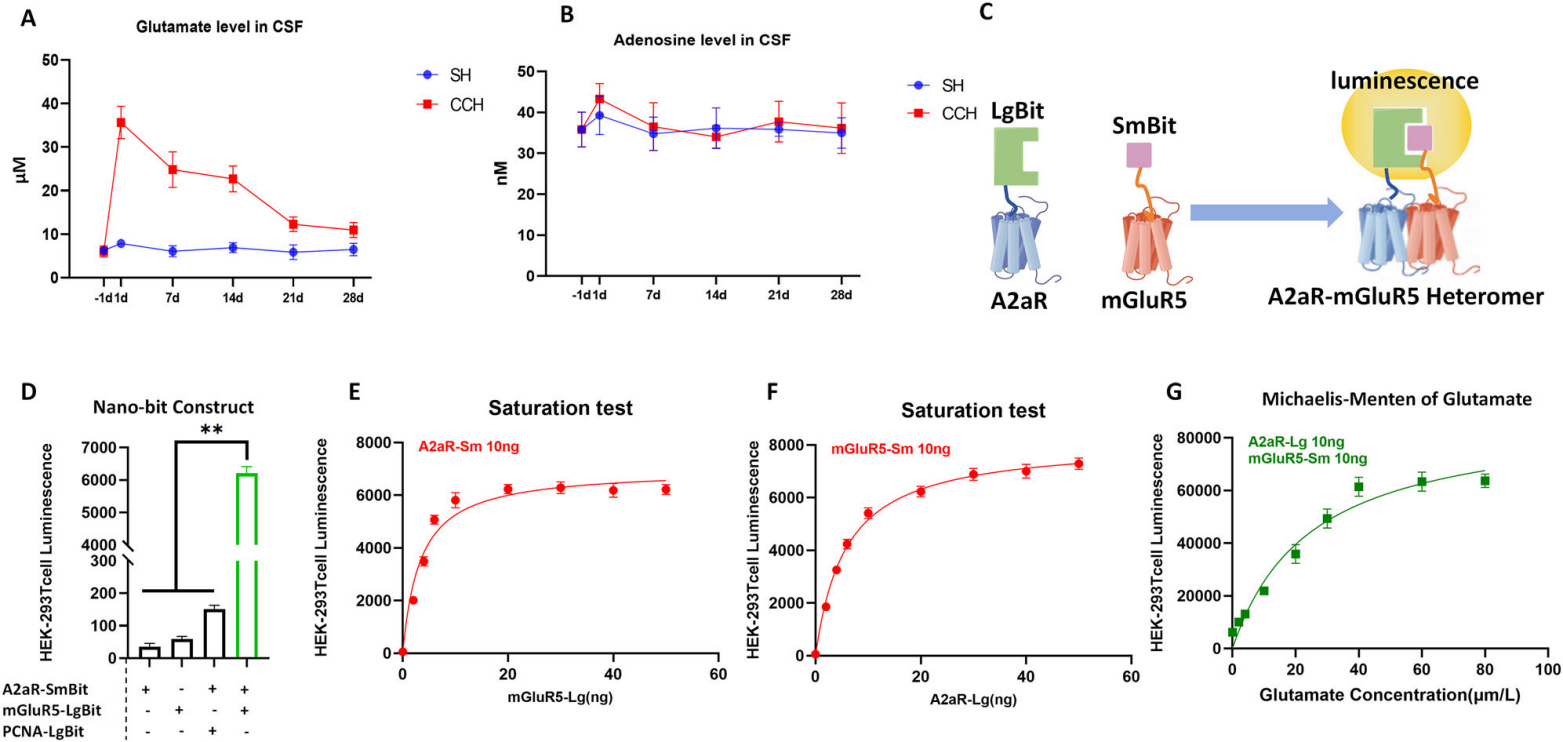
High glutamate concentration promotes the formation of A2aR–mGluR5 heteromer.

Previous studies have indicated that the synergistic effect of mGluR5 and A2aR may be a critical mechanism through which brain glial cells participate in proinflammatory responses, with glutamate identified as a key mediator of this mechanism. To investigate this further, we first measured glutamate and adenosine levels in the brains of CCH model rats from Week 1 to Week 4. The results showed that glutamate levels were significantly elevated during Weeks 1 and 2 compared with the −1 d group (representing the baseline level), followed by a decline starting in Week 3, and a return to normal levels by Week 4, demonstrating an overall decreasing trend (Fig. 4*A*), and there was no significant difference in the adenosine levels at each time point from 1 to 4 W compared with the −1 d group(representing the baseline level; Fig. 4*B*). This may be related to the highly efficient inactivation mechanism of adenosine in the brain.

To explore the effect of glutamate levels on the formation of A2aR–mGluR5 heteromers, we employed the NanoBiT system. This system comprises two artificially recombinant subunits, LgBiT (18 kDa) and SmBiT (11 amino acids), which can be expressed as fusion proteins with two target proteins. When these proteins approach or form heteromers, the subunits bind to each other, forming an active enzyme that emits a bright luminescent signal in the presence of a substrate (Fig. 4*C*). HEK293ET cells were cotransfected with plasmids encoding mGluR5-SmBit and A2aR-LgBit. Three negative controls were established: SmBit-only, LgBit-only, and A2aR-LgBit cotransfected with PNCA-SmBit (PNCA served as a negative control since it is a nuclear protein that does not interact with A2aR). The luminescence signal of the mGluR5-SmBit and A2aR-LgBit system was two orders of magnitude higher than that of the controls (Fig. 4*D*). Saturation experiments further validated the specificity of the interaction, showing a typical saturation curve as the plasmid concentrations increased (Fig. 4*E*,*F*). These results confirmed the specific binding between A2aR and mGluR5.

We next evaluated the influence of glutamate on A2aR–mGluR5 heteromer formation. The luminescence signal of the A2aR–mGluR5 NanoBiT system increased significantly with rising glutamate concentrations and eventually plateaued (Fig. 4*G*). These findings suggest that high glutamate concentrations promote the formation of A2aR–mGluR5 heteromers.

### High glutamate concentration promotes the formation of A2aR–mGluR5 heteromer in microglia and mediates the polarization of microglia to M1 phenotype

To further explore the effects of A2aR–mGluR5 heteromer formation on microglia, we conducted primary microglia experiments under hypoxic conditions. The experimental groups included the following:

1. **A:** Blank group
2. **B:** 20 μM glutamate
3. **C:** 40 μM glutamate
4. **D:** Hypoxia (1% O_2_, 24 h)
5. **E:** Hypoxia + 20 μM glutamate
6. **F:** Hypoxia + 40 μM glutamate

**Figure 5.**
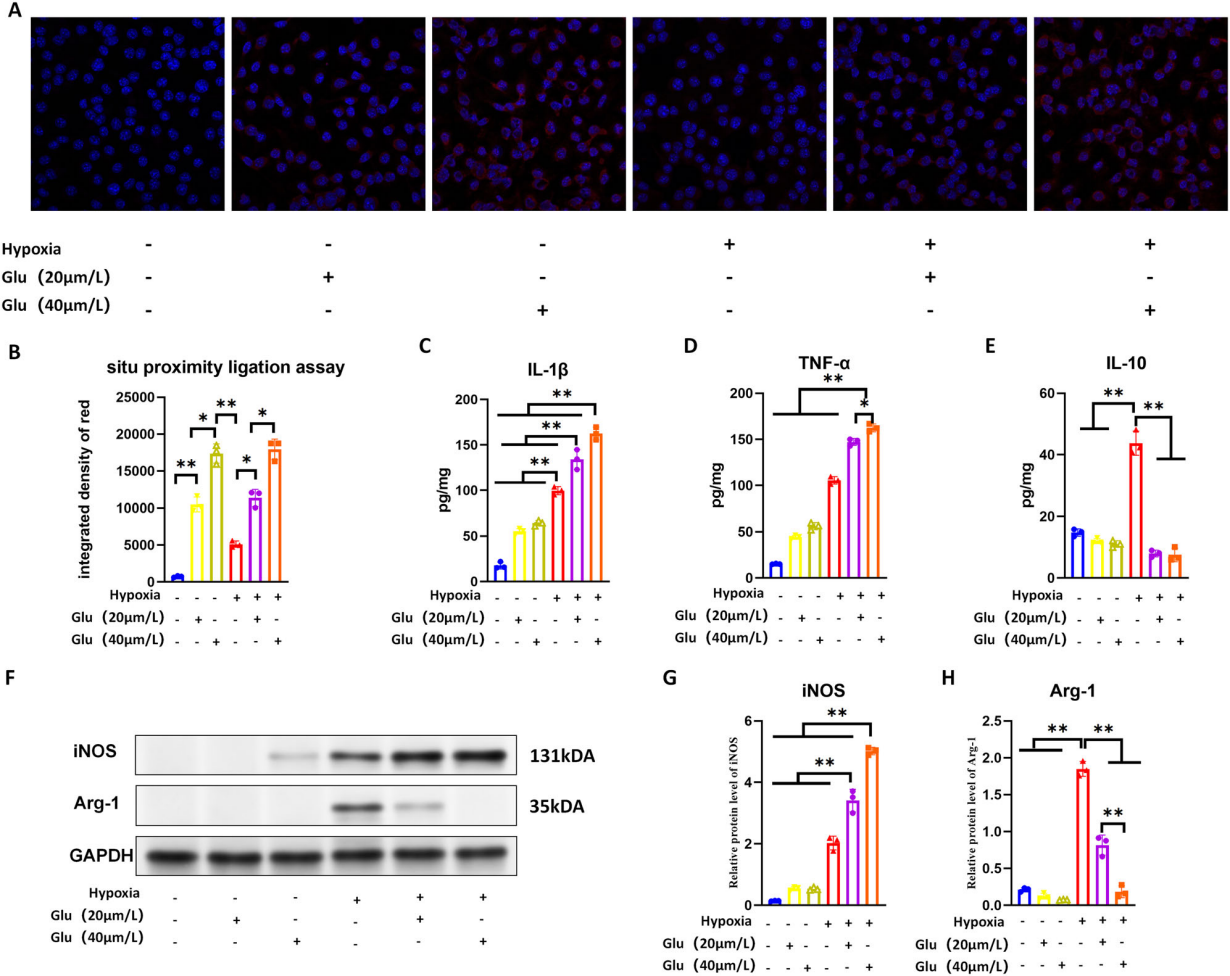
High glutamate concentration promotes the formation of A2aR–mGluR5 heteromer in microglia and mediates the polarization of microglia to M1 phenotype.

In situ PLA revealed significantly enhanced fluorescent intensity in Groups B, C, E, and F compared with the blank control Group A. Among these, Groups C and F (40 μM glutamate) showed significantly stronger fluorescent intensity than Groups B and E (20 μM glutamate; Fig. 5*A*,*B*). These results confirmed a positive correlation between A2aR–mGluR5 heteromer formation and glutamate concentration.

ELISA results demonstrated that proinflammatory cytokines IL-1β and TNF-α were significantly elevated in Group D compared with the blank Group A. Further increases were observed in Groups E and F, with the degree of elevation positively correlated with glutamate concentration. Anti-inflammatory cytokine IL-10 levels were significantly higher in Group D compared with the blank group, with no significant differences among the other groups (Fig. 5*C–E*). WB analysis showed that iNOS expression was significantly increased in Group D compared with the blank Group A and further elevated in Groups E and F, proportional to glutamate concentration. Conversely, Arg-1 expression was significantly higher in Group D compared with Group A but decreased in Groups E and F, with the reduction proportional to glutamate concentration (Fig. 5*F*–*H*).

These findings suggest that hypoxia induces microglial activation and polarization, with both M1 and M2 phenotypes present but predominantly M1. High glutamate concentrations under hypoxia shift microglial polarization toward the proinflammatory M1 phenotype. Based on these results, we hypothesize that elevated glutamate levels in the early stages of the CCH model promote A2aR–mGluR5 heteromer formation in microglia, driving polarization toward the proinflammatory M1 phenotype.

### A2aR–mGluR5 heteromer formation alters A2aR signaling pathway bias: a2aR monomer activation promotes microglial polarization to M2 phenotype through the Gs-mediated PKA pathway, while A2a–mGluR5 heteromer formation mediated by glutamate concentration changes promotes microglial polarization to proinflammatory phenotype through the Gq-mediated PKC pathway

**Figure 6.**
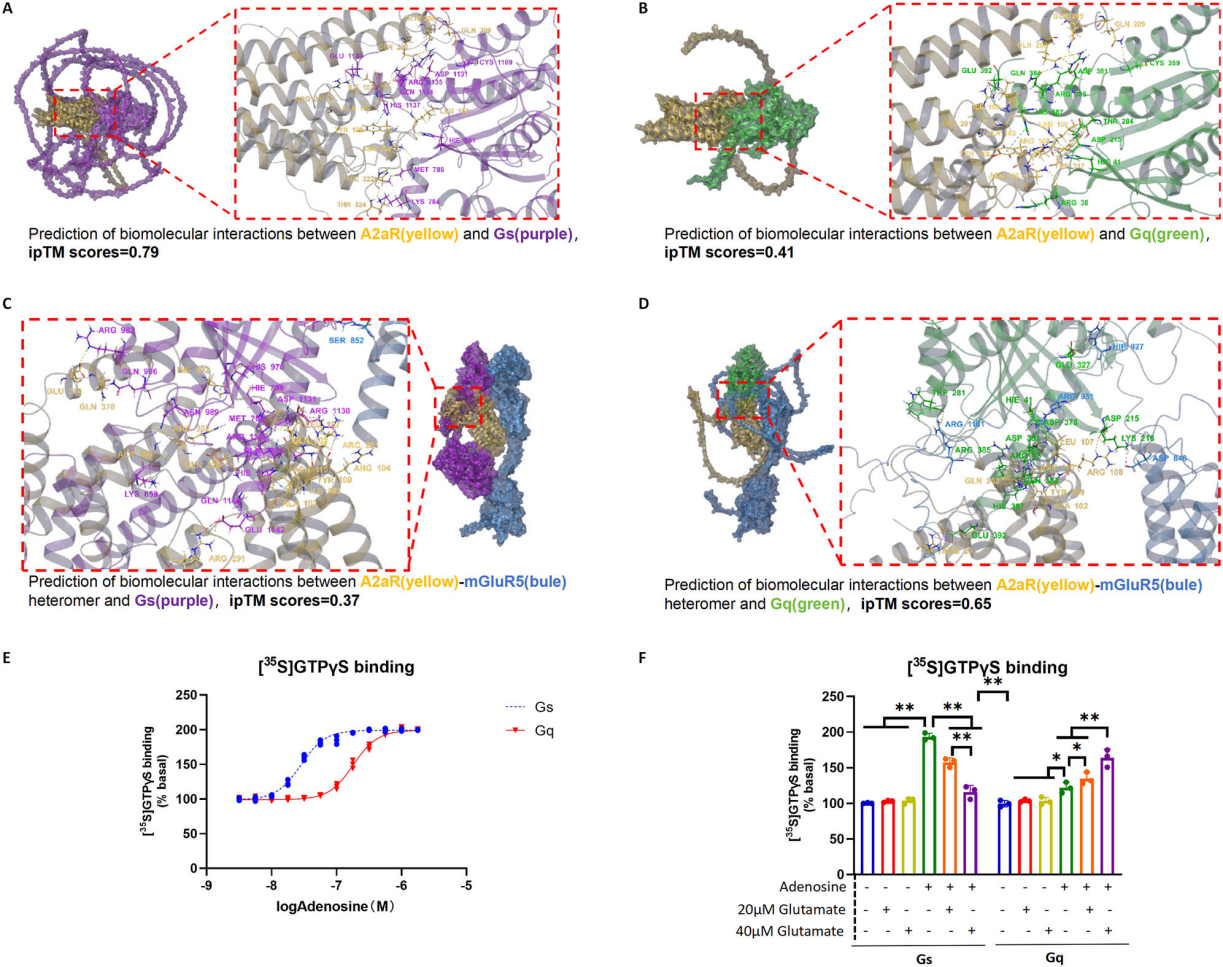
A2aR–mGluR5 heteromer formation alters A2aR signaling pathway bias.

A2aR is a Class A GPCR that binds to different subunits of G-protein and activates corresponding downstream pathways. Previous studies have shown that Gs and Gq subunits of G-protein are the major G-protein–binding subunits of A2aR (Bennett et al., 2013). To explore the effect of A2aR–mGluR5 heteromer formation on A2aR pathway bias, we predicted the affinity of the A2aR monomer state and A2aR–mGluR5 heteromer state for the two G-protein subunits, respectively, using the AlphaFold technique. In the AlphaFold prediction results, the interface prediction template modeling (ipTM) score is derived from a measure called the template modeling (TM) score, which measures the accuracy of the entire prediction structure. It can be assumed that the higher the ipTM score, the higher the binding propensity of the two subunits (Abramson et al., 2024). The predicted results showed that A2aR monomer had a higher binding affinity with Gs (ipTM, 0.79) than with Gq (ipTM, 0.41; Fig. 6*A*,*B*), while A2aR–mGluR5 heteromer had a higher binding affinity with Gq (ipTM, 0.65) than with Gs (ipTM, 0.37; Fig. 6*C*,*D*).

To investigate the difference in downstream signaling between A2aR monomers and A2aR–mGluR5 heteromers, we prepared crude membranes from primary microglia and performed the [^35^S]GTPγS binding assays followed by immunoprecipitation of Gs or Gq proteins. Adenosine-activated Gs and Gq proteins in primary microglia membranes with an EC50 of 31.62 ± 1.36 and 177.79 ± 5.54 nM, respectively (Fig. 6*E*). The results also showed that the addition of glutamate reduced the binding rate of Gs and increased the binding rate of Gq, and the degree of change was proportional to the glutamate concentration (Fig. 6*F*).

To investigate the role of A2aR–mGluR5 heteromer formation in microglial polarization, primary rat microglia were isolated for in vitro experiments. The groups were as follows:

1. **A:** Blank control
2. **B:** Hypoxia control
3. **C:** Hypoxia + CGS21680 (A2aR agonist)
4. **D:** Hypoxia + CGS21680 + H89 (PKA inhibitor)
5. **E:** Hypoxia + glutamate + CGS21680
6. **F:** Hypoxia + glutamate + CGS21680 + Go 6983 (PKC inhibitor)

**Figure 7.**
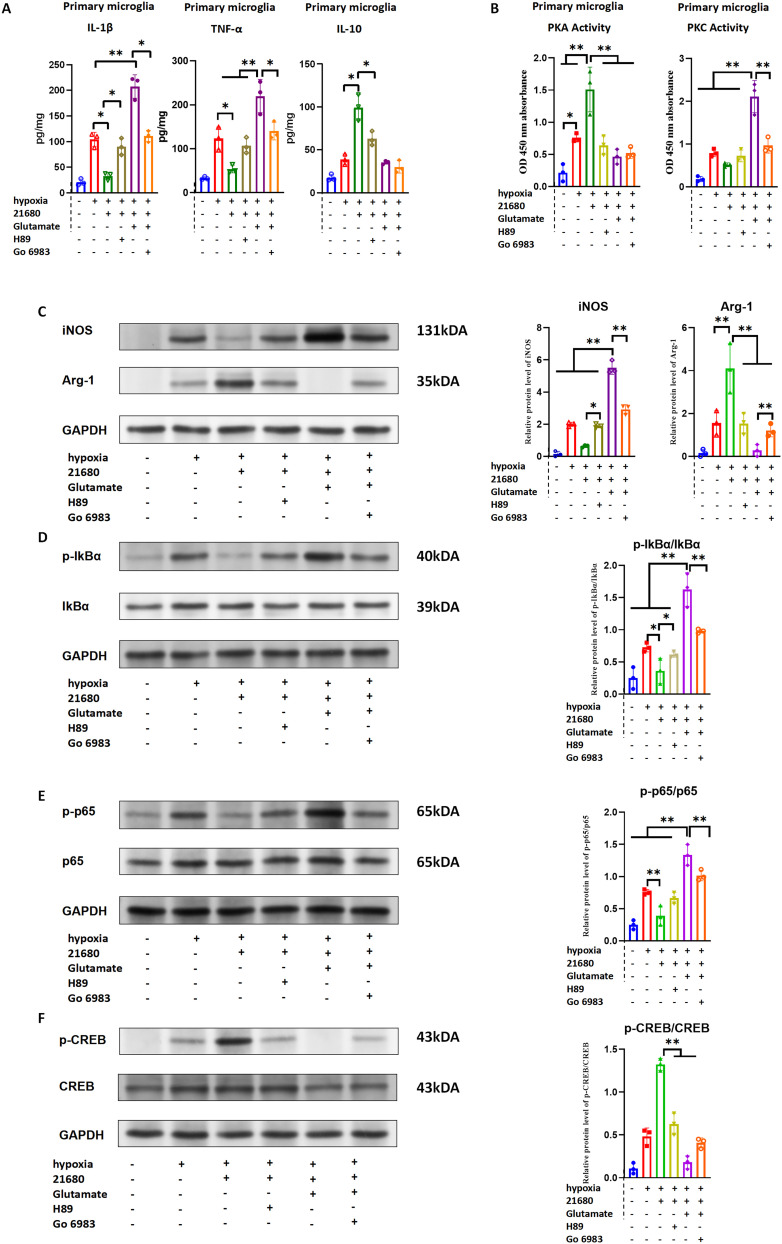
A2aR monomer activation promotes microglial polarization to M2 phenotype through Gs-mediated PKA pathway, while A2aR–mGluR5 heteromer formation mediated by glutamate concentration changes promotes microglial polarization to proinflammatory phenotype through the Gq-mediated PKC pathway.

ELISA results showed significant increases in proinflammatory factors (IL-1β and TNF-α) and anti-inflammatory factor (IL-10) in Group B (hypoxia) compared with Group A (blank control). Group C exhibited significantly reduced IL-1β and TNF-α and increased IL-10 levels compared with Group B, suggesting a protective role of CGS21680 under hypoxic conditions. The addition of H89 (Group D) reversed these protective effects, as indicated by increased IL-1β and TNF-α levels and decreased IL-10 levels. In Group E (CGS21680 + high glutamate), IL-1β and TNF-α levels were significantly elevated, and IL-10 levels were reduced compared with Groups B and C, indicating a reversal of the protective effects. In Group F (PKC inhibitor Go 6983 added), IL-1β and TNF-α levels decreased compared with Group E, although IL-10 levels remained unchanged, suggesting that PKC inhibition mitigated the damaging effects of the glutamate-CGS21680 combination (Fig. 7*A*).

WB results revealed that iNOS and Arg-1 levels increased in Group B compared with Group A. Group C exhibited decreased iNOS and increased Arg-1 expression compared with Group B, while these effects were reversed in Group D. In Group E, iNOS expression was significantly elevated, and Arg-1 expression was reduced compared with Groups B, C, and D. In Group F, iNOS levels decreased, and Arg-1 levels increased compared with Group E (Fig. 7*C*). These results suggest that hypoxia-induced microglial polarization predominantly to the M1 phenotype, with CGS21680 polarizing microglia toward the M2 phenotype under hypoxia. This polarization to M2 was reversed by PKA inhibition, while glutamate in combination with CGS21680 promoted M1 polarization, which was reversed by PKC inhibition.

Mechanistic investigations demonstrated that hypoxia combined with CGS21680 activated A2aR monomers, increasing *p*-CREB/CREB phosphorylation, PKA activity, and Arg-1 expression, which were reversed by PKA inhibition (Fig. 7*B*,*C*,*F*). In contrast, hypoxia with CGS21680 and glutamate activated A2aR–mGluR5 heteromers, increasing PKC-related protein phosphorylation (*p*-p65/p65, *p*-IkBα/IkBα) and iNOS expression, which were reversed by PKC inhibition (Fig. 7*B*–*E*). These findings indicate that A2aR monomer activation promotes M2 polarization through the PKA pathway, while glutamate-mediated A2aR–mGluR5 heteromer formation promotes M1 polarization through the PKC pathway.

### The combination of glutamate release inhibitor (riluzole) and A2aR agonist exerts a protective effect by promoting the polarization of microglia to M2 phenotype in the CCH model

**Figure 8.**
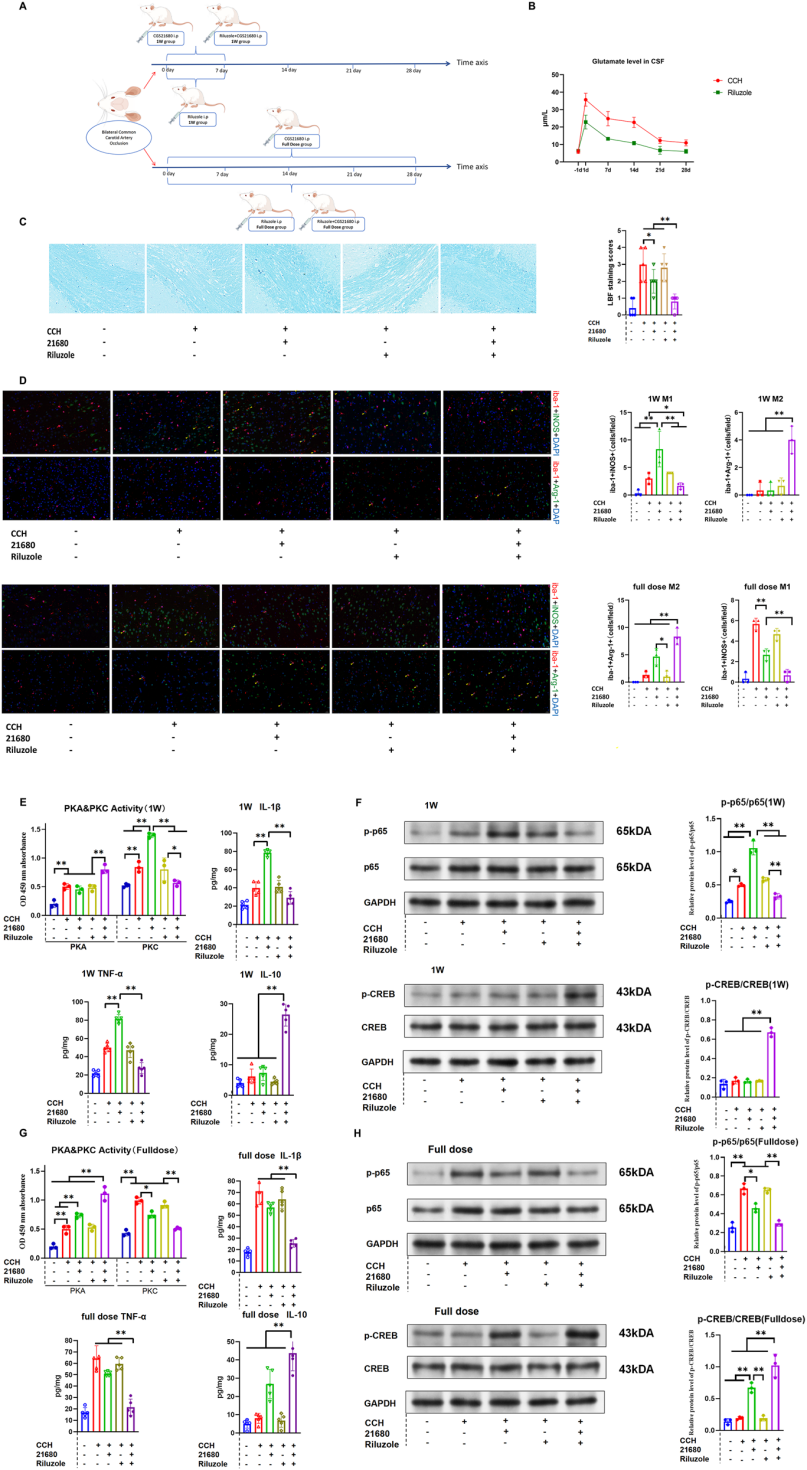
The combination of glutamate release inhibitor (riluzole) and A2aR agonist exerts a protective effect by promoting the polarization of microglia to the M2 phenotype in the CCH model.

The previous findings were validated in an animal model. First, the effect of the glutamate release inhibitor riluzole on brain glutamate levels in CCH was confirmed. Riluzole[Fig eN-NWR-0579-24F9] significantly reduced brain glutamate levels from 1 to 4 weeks after CCH induction (Fig. 8*B*).

**Figure 9. eN-NWR-0579-24F9:**
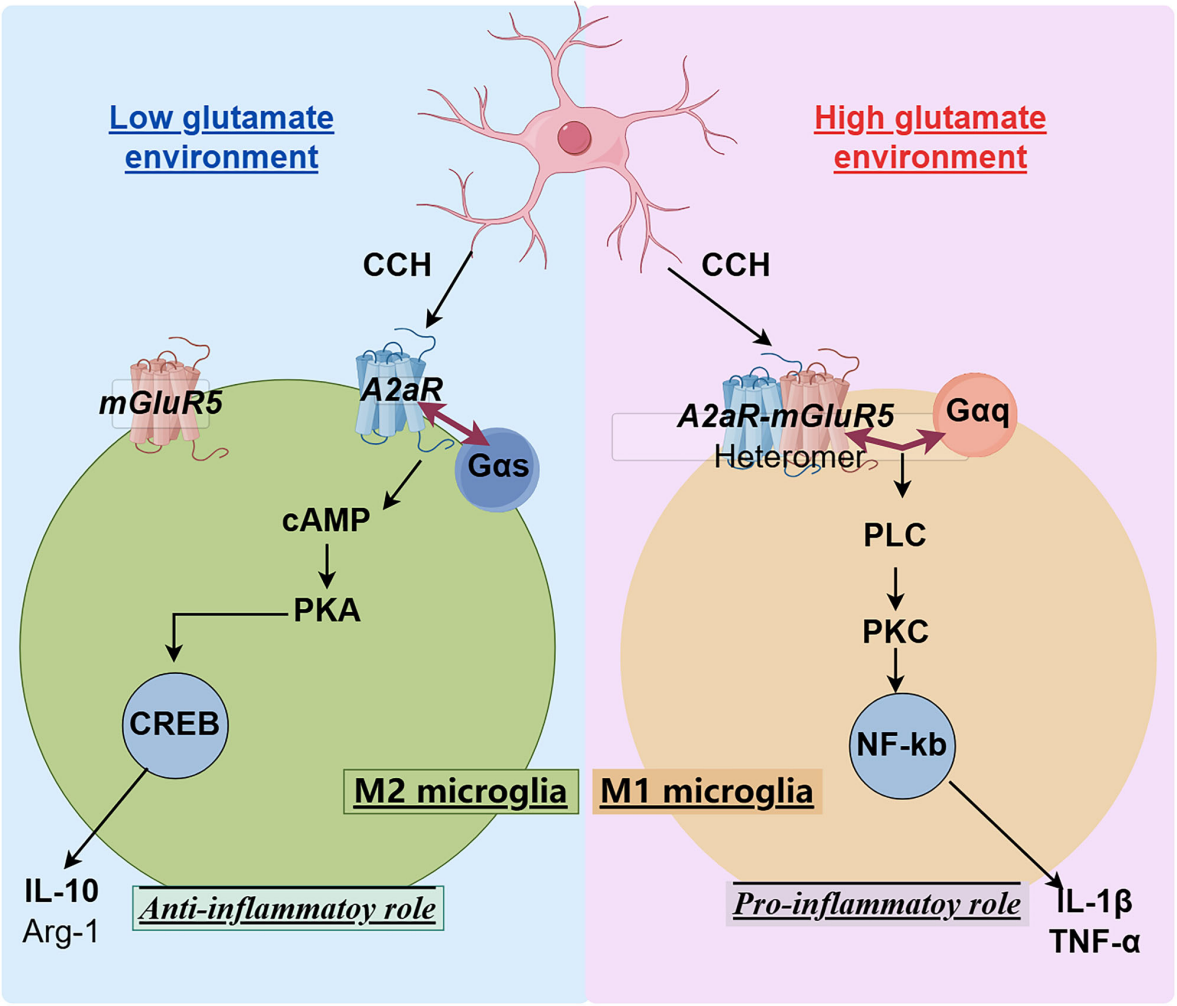
A graphical depiction of the dual role of A2aR in CCH.

Rats were divided into five groups as follows:

1. **A:** SH group
2. **B:** CCH group
3. **C:** CCH + A2aR agonist
4. **D:** CCH + riluzole
5. **E:** CCH + A2aR agonist + riluzole (Fig. 8*A*)

At 1 week post-treatment, IL-1β, TNF-α levels, PKC activity, and PKC-related protein phosphorylation (*p*-p65/p65) in Group C were significantly elevated compared with Group B (Fig. 8*E*,*F*), with increased M1 microglia (iba-1 and iNOS double-positive; Fig. 8*D*). This indicates that A2aR agonists promoted M1 polarization by upregulating the PKC pathway during the early stages. Group E showed reduced IL-1β TNF-α levels, PKC activity, PKC-related protein phosphorylation (*p*-p65/p65), increased IL-10 levels, PKA activity, PKA-related protein phosphorylation (*p*-CREB/CREB), and elevated M2 microglia (iba-1 and Arg-1 double–positive) compared with Group C (Fig. 8*D*–*F*), suggesting that the combination of riluzole and A2aR agonist reversed the proinflammatory effects by upregulating the PKA pathway.

At 4 weeks post-treatment (full dose), Group C showed decreased IL-1β, TNF-α levels, PKC activity, and PKC-related protein phosphorylation (*p*-p65/p65) while increased IL-10 levels, PKA activity, PKA-related protein phosphorylation (*p*-CREB/CREB), and higher M2 microglia counts compared with Group B (Fig. 8*G*,*H*,*D*), along with reduced white matter damage scores on LFB staining (Fig. 6*C*). Group E exhibited further reductions in IL-1β, TNF-α levels, PKC activity, and PKC-related protein phosphorylation (*p*-p65/p65) while increased IL-10 levels, PKA activity, PKA-related protein phosphorylation (*p*-CREB/CREB), higher M2 microglia counts (Fig. 8*G*,*H*,*D*), and improved LFB scores compared with group C. These results suggest that the A2aR agonist exerted anti-inflammatory effects by upregulating the PKA pathway over a 4 week treatment period, and combining riluzole with the A2aR agonist enhanced these effects.

## Discussion

The pathological mechanism of CCH white matter lesions involves neuroinflammation, oxidative stress, mitochondrial dysfunction, and lipid metabolism disorders (Washida et al., 2019). Among these, microglia-mediated neuroinflammation is widely recognized as a central driver in the progression of CCH white matter lesions (Long et al., 2024). Microglia, which perform functions analogous to peripheral macrophages, are highly heterogeneous and exhibit significant activation in central nervous system (CNS) diseases. They are classically categorized into M1 (proinflammatory) and M2 (anti-inflammatory) phenotypes, which regulate neuroinflammation (Fermi et al., 2023; Liu et al., 2024). Despite the existence of diverse microglial subtypes across various pathophysiological processes, the M1/M2 classification remains a practical framework for understanding the pro- and anti-inflammatory roles of microglia. Modulating the M1/M2 phenotype balance is a critical strategy for mitigating neuroinflammatory conditions (Long et al., 2024).

In this study, microglial activation was observed within 4 weeks following BCCAO surgery, predominantly polarizing toward the M1 phenotype. This activation correlated with elevated levels of proinflammatory cytokines IL-1β and TNF-α, demyelination of cerebral white matter, and structural damage to nerve fibers.

The A2aR is a key modulator in various neurological disorders (Karati et al., 2023), particularly in the context of neuroinflammation (Prasad et al., 2024). Microglia serve as primary effector cells through which A2aR regulates neuroinflammatory processes, and A2aR is intimately associated with microglial activation and polarization (Orr et al., 2009; Dai and Zhou 2011). However, its precise role and underlying mechanisms in neuroinflammation remain incompletely understood. Depending on the pathological state and microenvironment, A2aR exhibits dichotomous roles, functioning as either “anti-inflammatory” or “proinflammatory.”

Using a validated CCH model, we performed BCCAO surgery on 12-week-old male SD rats following a classical method (Wang et al., 2024). Doppler ultrasonography confirmed that BCCAO effectively reduced CBF from Weeks 1–4 postsurgery, with stable results. Subsequent experiments demonstrated that activating A2aR with the agonist CGS21680 during Weeks 1–2 post-BCCAO facilitated microglial polarization toward the M1 phenotype, exerting a proinflammatory effect. Conversely, A2aR activation during Weeks 3–4 promoted polarization toward the M2 phenotype, leading to an anti-inflammatory effect. These findings indicate that A2aR assumes opposite roles in neuroinflammation at different stages of the CCH model.

The structural configuration of molecules dictates their functional roles. A2aR's divergent functions at various stages post-BCCAO may be attributed to dynamic changes in the postoperative microenvironment and interactions with other molecular entities that alter receptor conformation. Using Co-IP–MS, we identified mGluR5 as the A2aR-binding protein with the highest signal intensity and iBAQ value. As a G-protein–coupled membrane receptor, mGluR5 likely interacts with A2aR, potentially altering its configuration.

To elucidate their interaction, we employed AlphaFold3 technology to predict the protein structures of A2aR and mGluR5. Our analysis revealed that TYR175, GLN202, and ARG194 of A2aR formed three hydrogen bonds with VAL788, LYS676, and LYS759 of mGluR5, respectively (Fig. 3*E*,*F*). These findings suggest a strong interaction propensity between A2aR and mGluR5, supporting the likelihood of their existence as heteromers.

The presence of A2aR–mGluR5 heteromers was confirmed through WB, Co-IP, and immunofluorescent staining. WB results indicated no significant changes in A2aR and mGluR5 expression levels 4 weeks post-BCCAO surgery (Fig. 3*G*). Co-IP results demonstrated Co-IP of mGluR5 with anti-A2aR antibodies and A2aR with anti-mGluR5 antibodies in primary microglia exposed to chronic hypoxia (Fig. 3*H*). Additionally, triple immunofluorescent staining revealed colocalization of iba-1, A2aR, and mGluR5 in the white matter regions (Fig. 3*J*). These results collectively confirm the presence of A2aR–mGluR5 heteromers in microglia.

mGluR5 is a metabotropic glutamate receptor (MGR) that belongs to the Gq family of GPCRs. MGRs are subdivided into three groups: Group I (mGluR1 and mGluR5), Group II (mGluR2 and mGluR3), and Group III (mGluR4, mGluR6, mGluR7, and mGluR8). These receptors are abundantly expressed in the CNS and primarily regulate the slow biological effects of glutamate. Group I receptors, such as mGluR5, mediate slow excitatory responses through calcium ion mobilization and PKC activation. In contrast, Groups II and III, belonging to the Gαi/o class, mediate slow inhibitory effects primarily by inhibiting PKA activity (Niswender et al., 2005; Niswender and Conn 2010).

Using single-molecule total internal reflection fluorescent microscopy, previous studies revealed that mGluR5 forms heteromers with various GPCRs, and these heteromeric interactions are in dynamic equilibrium (Kasai et al., 2011; Calebiro and Sungkaworn 2018). Dai et al. (2013) demonstrated that mGluR5 forms heterodimers with A2aR, reversing the anti-inflammatory effects of A2aR. These findings align with the results of our study, wherein we found that receptor structural configurations, modulated by ligand interactions, influence receptor dimerization and polymerization processes.

Glutamate, the most abundant excitatory neurotransmitter in the CNS and the natural ligand of mGluR5, plays a pivotal role in receptor interactions. Previous research has shown that elevated glutamate levels promote the formation of A2aR–mGluR5 heteromers on neutrophils (Dai et al., 2013). Adenosine, as the major ligand of A2aR, is highly correlated with brain energy metabolism (Chen et al., 2018). In our study, we used microdialysis combined with HPLC-MS to monitor glutamate and adenosine concentrations in the brains of rats following BCCAO surgery. The results indicated that there was no significant difference in the adenosine levels at each time point from −1 d to 4 weeks compared with the sham group. This may be related to the highly efficient inactivation mechanism of adenosine in the brain, and glutamate levels were significantly elevated during Weeks 1–2 postsurgery compared with the sham group. By Week 3, glutamate levels began to decline, and by Week 4, they returned to near-normal levels. These results suggest that glutamate may be closely related to the formation of A2aR–mGluR5 heteromer under CCH.

To further investigate the relationship between glutamate concentrations and A2aR–mGluR5 heteromer formation, we utilized the NanoBiT system. This system consists of two recombinant subunits, LgBiT and SmBiT, which produce a luminescent signal when the target proteins (mGluR5 and A2aR) form a heteromer. Our results showed that the luminescence signal of the A2aR–mGluR5 system was two orders of magnitude higher than that of the negative controls, confirming the existence of A2aR–mGluR5 heteromers under physiological conditions. Additionally, increasing glutamate concentrations in the system significantly enhanced luminescence, demonstrating that high glutamate levels promote A2aR–mGluR5 heteromer formation.

To evaluate the functional effects of A2aR–mGluR5 heteromers on microglia, we conducted ex vivo experiments using primary microglia under hypoxic conditions. PLA results showed that A2aR–mGluR5 heteromer levels increased with rising glutamate concentrations in microglia. WB and ELISA analyses revealed that hypoxia-induced microglial activation resulted in both M1 and M2 phenotypes, with the M1 phenotype predominating. High glutamate concentrations further shifted microglial polarization toward the proinflammatory M1 phenotype.

Based on these findings, we hypothesize that elevated glutamate levels in the early stages of CCH facilitate A2aR–mGluR5 heteromer formation in microglia, thereby driving the transformation of microglia toward the proinflammatory M1 phenotype.

In recent years, research on GPCR heteromers has revealed that GPCR monomers, homodimers, heteromers, and heteromultimers serve as distinct functional entities, each with unique pharmacological properties and signal transduction pathways (Franco and Navarro 2023). Several studies have demonstrated that GPCR heteromerization mediates biased signaling, altering or even reversing the functions of GPCR monomers. For instance, Bai et al. (2014) reported that the formation of apelin receptor (APJ)-B1R heteromers reduces Gi pathway activation while promoting Gq-mediated signaling. Similarly, Harmeier et al. (2015) showed that D2R-TAAR1 heteromerization attenuates cAMP signaling while enhancing β-arrestin2 signaling and reducing GSK3β activation. These findings underscore the capacity of GPCR heteromerization to modulate downstream signaling selectivity, providing novel insights into receptor functionality and therapeutic potential.

A2aR, a Class A GPCR widely distributed in the brain tissue, can couple with various G-protein subtypes such as Gs and Gq (Bennett et al., 2013). Among these pathways, the Gs/cAMP/PKA pathway is widely considered the most critical for A2aR. When activated, A2aR binds to Gs, increasing cAMP levels, which enhances PKA activity and leads to the phosphorylation of the CREB, initiating downstream reactions (Wu et al., 2021; Sun et al., 2022). CREB activation has been extensively documented, playing essential roles in neuronal protection, synaptic regeneration, and memory formation. Additionally, studies have shown that the CREB pathway promotes the transformation of macrophages into the M2 phenotype (Ruffell et al., 2009; Zhang et al., 2016). Conversely, previous findings have reported that when A2aR interacts with mGluR5, the PKA pathway is inhibited, and the PKC pathway is activated via Gq (Dai et al., 2013).

To explore the pathway bias induced by A2aR–mGluR5 heteromer formation and its functional alterations, we used AlphaFold3 to predict the affinity of the heteromer with Gs and Gq. The results showed that A2aR monomer had higher affinity for Gs than for Gq, whereas the A2aR–mGluR5 heteromer exhibited higher affinity for Gq than for Gs. [^35^S]GTPγS binding assay also indicated that A2aR monomer on primary microglia cell membrane was more likely to bind to Gs, and when glutamate concentration was increased, A2aR on primary microglia cell membrane was more likely to bind to Gq in the form of A2aR–mGlur5 heteromer (Fig. 6). These results suggest that the change of A2aR from monomer to A2aR–mGluR5 heteromer leads to the change of its binding G-protein α subunit from Gs to Gq, which in turn leads to the change of the downstream dominant pathway from PKA to PKC. We further performed in vitro experiments using primary microglia. Under hypoxic conditions, A2aR activation alone promoted microglial polarization to the M2 phenotype via the cAMP/PKA/CREB signaling pathway, an effect inhibited by a PKA inhibitor. Conversely, at elevated glutamate concentrations, substantial A2aR–mGluR5 heteromer formation occurred. In this context, A2aR activation promoted microglial polarization to the proinflammatory M1 phenotype via the PKC pathway, an effect reversed by a PKC inhibitor. These findings indicate that A2aR–mGluR5 heteromer formation alters the downstream signaling pathway bias of the A2aR monomer. Specifically, A2aR monomer activation triggers the PKA/CREB pathway through Gs, promoting M2 polarization. In contrast, A2aR–mGluR5 heteromer activation directs signaling through the Gq/PKC/p65 pathway, favoring M1 polarization and proinflammatory effects (Fig. 7).

GPCR heteromerization is a critical regulatory mechanism of GPCR function (Farran 2017), with significant therapeutic potential for CNS disorders (Conn et al., 2014). For instance, Blasiak et al. (2017) reported significant differences in Ca2+ levels activated by the D1R–D2R heterodimer compared with the D2R monomer, suggesting its potential as a novel drug target for Alzheimer's disease. Similarly, Kwan's research (Kwan et al., 2021) demonstrated that the 5-HT2AR–GluR2 heteromer modulates the pharmacological effects of hallucinogenic and antipsychotic agents. Combined GluR2 agonists and 5-HT2AR inhibitors potentiated the antidyskinesia and antipsychotic effects of 5-HT2AR blockade.

In this study, we explored targeting the A2aR–mGluR5 heteromer as a therapeutic strategy for CCH-associated neuroinflammation. Specifically, we employed the glutamate inhibitor riluzole to attenuate glutamate levels following BCCAO surgery, aiming to inhibit A2aR–mGluR5 heteromer formation. Our results revealed that the glutamate level in the brain of CCH model was significantly increased in the early stage (1–2 weeks), and riluzole could reduce the glutamate level of CCH model to the level of the SH group. Administration of an A2aR agonist increased proinflammatory factors and promoted M1 polarization in CCH model by upregulating the PKC pathway. Conversely, riluzole mitigated the proinflammatory effects of the A2aR agonist, shifting microglial polarization toward the M2 phenotype by upregulating the PKA pathway. After 4 weeks of treatment, A2aR agonist administration increased anti-inflammatory factors and M2 polarization by upregulating the PKA pathway. Combining riluzole further enhanced these anti-inflammatory effects (Fig. 8). These results suggest that the dual effect of A2aR is closely related to the level of glutamate in the brain: When the brain is at a high glutamate level, A2aR exists in the form of A2aR–mGluR5 heteromer to promote the M1 polarization of microglia and play a proinflammatory role by activating the PKC pathway. When the brain is at a trough level of glutamate, A2aR exists in the form of monomer to promote the M2 polarization of microglia and play an anti-inflammatory role by activating the PKA pathway (Fig. 9). The results are consistent with the results of in vitro cell experiments.

The study has several limitations. The molecular mechanisms underlying A2aR–mGluR5 heteromer signaling in microglia under CCH conditions remain incompletely understood, largely due to the lack of detailed receptor structural information in different conformational states. Additionally, the mechanism by which elevated glutamate concentrations facilitate A2aR–mGluR5 heteromer formation requires further investigation. Future research will employ cryo-electron microscopy and other advanced techniques to elucidate the three-dimensional structure and formation mechanisms of the A2aR–mGluR5 heteromer.

In summary, this study confirmed microglial polarization as a central mechanism underlying white matter lesions in CCH neuroinflammation. Additionally, we identified a novel mechanism involving GPCR heteromerization, providing an experimental foundation for developing anti-inflammatory and reparative therapies targeting the A2aR–mGluR5 heteromer in CCH neuroinflammation.

### Data and materials availability

Any additional information related to this study is available from the author for correspondence upon reasonable request.

## Synthesis

Reviewing Editor: Jibran Khokhar, Western University Department of Anatomy and Cell Biology Decisions are customarily a result of the Reviewing Editor and the peer reviewers coming together and discussing their recommendations until a consensus is reached. When revisions are invited, a fact-based synthesis statement explaining their decision and outlining what is needed to prepare a revision will be listed below. The following reviewer(s) agreed to reveal their identity: NONE. Note: If this manuscript was transferred from JNeurosci and a decision was made to accept the manuscript without peer review, a brief statement to this effect will instead be what is listed below.

The authors have sufficiently addressed all the comments from the reviewers.
